# Aspirin versus placebo on estrogen levels in postmenopausal women: a double-blind randomized controlled clinical trial

**DOI:** 10.1186/s40360-022-00571-9

**Published:** 2022-05-17

**Authors:** Mohammad Bagher Oghazian, Nooshin Shirzad, Mahdi Ahadi, Shalaleh Eivazi Adli, Samaneh Mollazadeh, Mania Radfar

**Affiliations:** 1 Department of Internal Medicine, Faculty of Medicine, North Khorasan University of Medical Sciences, Bojnurd, Iran; 2 Endocrinology and Metabolism Research Institute, Tehran University of Medical Sciences, Tehran, Iran; 3 Department of Clinical Pharmacy, School of Pharmacy, Zanjan University of Medical Sciences, Zanjan, Iran; 4 School of Pharmacy and Pharmaceutical Sciences, Islamic Azad University of Tehran Medical Sciences, Tehran, Iran; 5 Natural Products and Medicinal Plants Research Center, North Khorasan University of Medical Sciences, Bojnurd, Iran; 6 Department of Clinical Pharmacy, Faculty of Pharmacy, Tehran University of Medical Sciences, Tehran, Iran

**Keywords:** Estradiol, Non-steroidal anti-inflammatory agents, Postmenopause, Sex hormone-binding globulin, Testosterone

## Abstract

**Background:**

Estrogen is involved in the pathogenesis of breast and gynecological cancers. Regular use of aspirin reduces estrogen levels. The present study aimed to evaluate the effect of aspirin on estrogen levels in postmenopausal women.

**Methods:**

This double-blind, placebo-controlled parallel-group trial was conducted on postmenopausal women referred to an outpatient clinic at a women’s hospital in Tehran. Volunteers were randomly assigned to receive aspirin 100 mg/day or placebo for 6 weeks. Estradiol, sex hormone-binding globulin (SHBG), and testosterone levels at baseline and at the end of the intervention were measured by ELISA. Data were analyzed using SPSS 20, Kolmogorov–Smirnov test, independent samples t-test, and Mann–Whitney U test.

**Results:**

Twenty-seven and 28 participants were finally analyzed in the aspirin and placebo groups, respectively. There was no significant difference between the two groups in body mass index (BMI), age, or menopausal years. There was a statistically significant difference (*p* = 0.002) in the amount of  change in estradiol levels of the intervention group (median=− 3.5 pg/ml) compared to the control group (median=1.5 pg/ml). In contrast, there were no significant differences between the two groups regarding testosterone and SHBG levels (*p* = 0.58, *p* = 0.32).

**Conclusions:**

Since low doses of aspirin may decrease estradiol levels, it could be considered a promising adjunctive therapeutic candidate in postmenopausal women to decrease BC incidence. However, further studies with larger sample sizes, measurements of estrogen levels and its related compounds in different time points accompanied by long-term follow-ups are needed to better elucidate the potential mechanisms by which nonsteroidal anti-inflammatory drugs (NSAIDs) negatively affect breast cancer.

**Trial registration:**

IRCT201012195397N1. Date of first registration: 03/01/2011.

## Background

Chronic inflammation is involved in the development and progression of several cancers [1, 2], including breast cancer (BC). Prior studies indicated that the use of nonsteroidal anti-inflammatory drugs (NSAIDs), such as aspirin, might be associated with a reduced risk of breast, ovarian, and endometrial cancer [3–6].

The chemopreventive effects of aspirin are mediated via suppression of cell proliferation, apoptosis, and modulation of lymphangiogenesis [7]. Although the exact mechanism through which aspirin exerts protective effects on BC is not fully understood, the primary mechanism could be explained by inhibiting cyclooxygenases (COXs), especially COX-2, followed by lower prostaglandin E-2 (PGE-2) synthesis. COX-2 overexpression in BC is associated with either poor prognosis and larger tumor size and lymph node metastasis [8]. Moreover, COX-2 can stimulate cytochrome P450 aromatase expression, which is responsible for converting androgen to estrogen, leading to increased estrogen production in adipose cells of breast tissue [9].

Regarding the unique role of aromatase as the source of estrogen production in postmenopausal women [10], aspirin consumption antagonizes the COX-2/PGE-2 signaling axis [11] and, consequently, might result in reduced estrogen production facilitated by suppressing aromatase activity [12].

Meta-analyses [3, 13, 14] and several studies [15] found that aspirin (or nonaspirin NSAIDs) use is associated with a reduced risk of breast cancer. In line with this, a dose-response meta-analysis [16] of cohort studies from approximately 1 million people concluded that the optimal aspirin dose for the chemoprevention of BC might be in the range of < 325 mg per day, 2–7 times/week, accompanied by long-term administration (> 5 years). Given the chemopreventive effects of aspirin in BC [17, 18] and the relations between high serum levels of steroids (estrogens and androgens) and increased BC risk in postmenopausal women [19–21], some studies were deliberated on introducing aspirin dosing to lower estrogen levels. However, these studies [17, 22, 23] and a randomized clinical trial (RCT) [11] did not succeed in concluding that the dose, frequency, and duration of aspirin use observed in the prevention of BC necessarily complied with those of estrogen lowering. Only one RCT has addressed the aspirin effects on estrogen levels in postmenopausal women [11], but it had no interest in the effects. Apart from that, no RCT has yet been designed to determine when estradiol and androgen levels initiate to decrease following aspirin administration. Therefore, we intended the administration of low-dose aspirin 100 mg/day vs. placebo for 6 weeks to assess the effect of a short time of aspirin use on estradiol (circulating estrogen) and sex hormone-binding globulin to measure free estradiol levels and testosterone (substrate for the aromatase enzyme) in postmenopausal women.

## Methods

The present study was a prospective, randomized, double-blinded, controlled parallel-group trial (ID: IRCT201012195397N1 registered on www.irct.ir) conducted between December 2019 and March 2021 and performed in the outpatient clinic at Arash Women’s Hospital, affiliated with Tehran University of Medical Sciences (TUMS). The study design and protocol were approved by the Institutional Review Board and the ethics committee of TUMS.

**Fig. 1:**
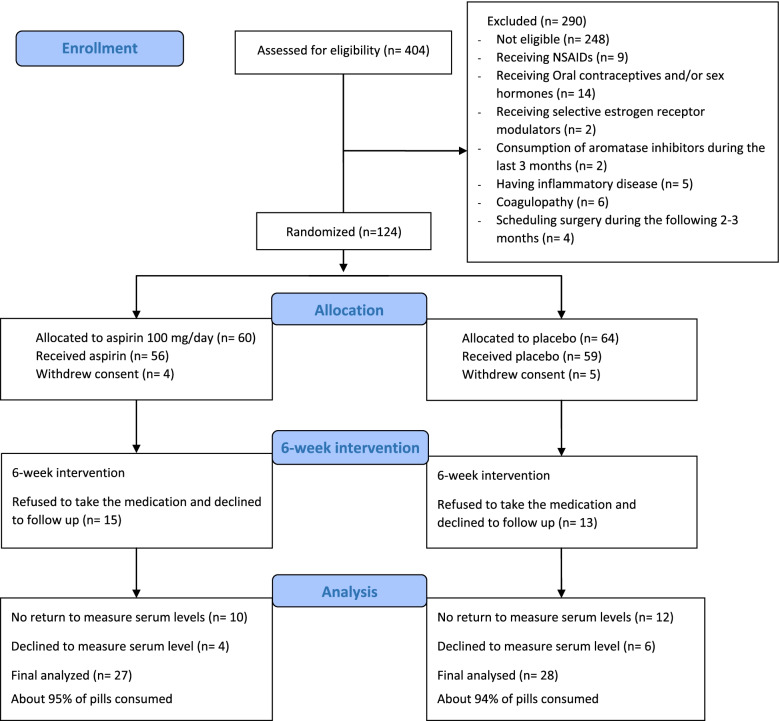
Consort flow diagram of study participants

Postmenopausal women referred to the outpatient clinic at the hospital were assessed to be eligible via medical history. A consort flow diagram of the study participants is shown in Fig. 1. Subjects who met the following inclusion criteria were enrolled in the study: aged between 45 and 85 years; no history of menstrual bleeding during the last 12 months; bilateral oophorectomy; and hysterectomy without bilateral oophorectomy (above 56 years in nonsmokers and 54 in smokers). In addition, the exclusion criteria were as follows: receiving NSAIDs and/or any anti-inflammatory drugs, sex hormones, oral contraceptives, selective estrogen receptor modulators, vaginal estrogen preparations or aromatase inhibitors during the last 3 months; inflammatory disease, coagulopathy complications, and surgery during the following 2–3 months.

Baseline blood samples were obtained from all participants to determine the serum levels of testosterone, estradiol, and SHBG. Participants were randomly allocated in a 1:1 ratio using the permuted-block randomization method to take aspirin (100 mg/day) or placebo orally for 6 weeks. Health care providers, participants, and data collectors were blinded to treatment assignment. At the end of the follow-up period, the serum levels of testosterone, estradiol, and SHBG were measured again by an ELISA reader (BioTek®, Synergy) based on the manufacturer’s manual instructions. It should be noted that the particular dosage of aspirin (100 mg/ml) was chosen according to the age and severity of gastrointestinal symptoms of the participants.

To explore the effect of aspirin on estradiol levels, we calculated 20 participants in each group by considering the mean difference of 5% (0.05) in estradiol levels between the intervention and placebo groups in case of a variance of 1% (0.01) with power = 80% and alpha error of 0.05. Once all the initial calculated number of participants completed the study (i.e., 40 participants), outcome adjudicators found that the study’s results did not have enough reliable power because the initial standard deviation (SD) for sample size calculation was considerably lower than the real obtained SD. Consequently, allocation to study groups continued to achieve targeted power. Outcome adjudicators announced when to terminate the study. They calculated the power of the study based on the collected data of the primary sample size and determined in a blinded setting how long the study would be continued. Since this study was a double-blinded trial, and we previously mentioned who was blinded; therefore, healthcare providers were blinded to the treatment assignment in all stages.

The distribution of continuous variables was analyzed by the Kolmogorov–Smirnov test. In addition, an independent samples t-test was used to analyze parametric data, whereas the Mann–Whitney U test was used to analyze significant differences in nonparametric data. Statistical analyses were performed by SPSS® Statistics software version 20.0, and a *p*-value < 0.05 was considered statistically significant.

## Results

### Inclusion and follow-up

In the current study, 124 women were randomly assigned to the aspirin or placebo groups. Of whom, 27 and 28 participants with mean (±SD) ages of 53.81 (± 4.46) and 53.43 (± 3.69) years were finally analyzed in the aspirin and placebo groups, respectively (Fig. 1).

### Baseline characteristics

Table 1 shows the characteristics and demographic data of the study’s participants. There was no significant difference between the two groups in body mass index (BMI), age, smoking history, and the number of years since menopause.

**Table 1:** Characteristics and demographics data of participants

Variable	Aspirin (***n*** = 27)	Placebo (***n*** = 28)
BMI (kg/m2)		
Mean ± SD	30.74 ± 2.51	28.84 ± 2.88
Range	26.16–35.30	23.31–35.00
Age (years)		
Mean ± SD	53.81 ± 4.46	53.43 ± 3.69
Range	46–61	47–61
Smoking	0	0
Number of years since menopause		
Median (IQR)	3.0 (2–4)	2.5 (2–3)
Range	1–5	1–6

### Main outcomes

A Wilcoxon signed-rank test revealed a significant difference between serum levels of estradiol at baseline and at the end of the sixth week in the aspirin group (Z = −2.55, *p* = 0.01). However, this significant difference was not seen in the placebo group (Table 2). In contrast, there were no statistically noticeable differences in the serum levels of testosterone and SHBG in the study groups, comparing baseline to the end of the sixth week.

**Table 2:** Changes in the serum markers at baseline and postintervention

Serum Levels of Markers	Aspirin group			Placebo group			Aspirin vs. placebo		
	Baseline	At the end of the intervention period	*p*-value^*^	Baseline	At the end of the intervention period	*p*-value^*^	At the end of the intervention period Aspirin	At the end of the intervention period Placebo	*p*-value^**^
Estradiol (pg/mL) Median (IQR)	15.00 (12.00–20.90)	12.70 (8.50–16.60)	0.01*	15.00 (11.35–22.25)	16.50 (11.30–22.10)	0.12	12.70 (8.50–16.60)	16.50 (11.30–22.10)	< 0.01
Testosterone (ng/dL) Median (IQR)	0.50 (0.40–0.80)	0.50 (0.40–0.70)	0.51	0.40 (0.40–0.60)	0.40 (0.40–0.60)	0.10	0.50 (0.40–0.70)	0.40 (0.40–0.60)	0.58
SHBG (nmol/L) Median (IQR)	26.30 (17.50–36.00)	26.00 (19.70–33.70)	0.80	26.30 (14.32–33.50)	25.20 (13.42–28.27)	0.08	26.00 (19.70–33.70)	25.20 (13.42–28.27)	0.33

*SHBG* Sex Hormone Binding Globulin

* Wilcoxon signed-rank test

^**^ Mann–Whitney U test

At the end of the sixth week, using the Mann–Whitney U test, data analysis enlightened that the amount of change from baseline (i.e., the difference between baseline level and end of the sixth week level in each patient) in the estradiol levels of the intervention group (aspirin 100 mg/day) was significantly less than those of the placebo group (median=−3.5 pg/ml vs. 1.5 pg/ml, respectively; p= 0.002; results not shown in table 2). Nevertheless, no significant differences were found in testosterone (*p* = 0.58) and SHBG (*p* = 0.33) concentrations between the aspirin and placebo groups in postmenopausal women. These results indicate that the changes in the estradiol level are not influenced by testosterone and SHBG.

### Safety

The proportion of participants who developed gastrointestinal complications (i.e., bleeding and dyspepsia) was not significantly different between the aspirin and placebo groups [18 out of 56 participants vs. 16 out of 59 participants (32% vs. 27%), respectively; *p* = 0.52]. Among those participants analyzed in the aspirin and placebo groups, 4 and 2 had dyspepsia, respectively. According to their self-report, they occasionally experienced this complication, which did not lead to the aspirin or placebo intake interruption. Finally, we did not find any significant difference between respondents [55 (44.35%)] and non-respondents [69 (55.64%)] regarding clinical characteristics and baseline levels of steroids (data not shown).

## Discussion

Aspirin is an NSAID that decreases prostaglandin levels by inhibiting COX-1 and -2 [24–26]. Since COX is a prognostic factor of BC and upregulates this cancer [27, 28], selective inhibition of this factor could reduce estradiol production in breast cells, as evidenced by celecoxib administration [23, 29]. Additionally, regular use of NSAIDs could reduce BC risk [30]. Among different NSAIDs, frequent use of aspirin could be more effective in reducing the risk of BC [31–33]. In addition to the results of our study, there is a line of evidence that NSAIDs (including aspirin and ibuprofen) could significantly decrease circulating estradiol levels in postmenopausal women, followed by protection against BC [23].

The inhibitory effects of aspirin on COX-2 are increased at high doses, and also, anticancer effects of aspirin were initially suggested in higher doses when administered for the long term as once-daily dosing [26]. However, there are inconsistent findings across studies to conclude the dose, frequency, and duration of aspirin use in lowering estrogen levels [11, 17, 22, 23]. In a randomized trial [11], the effects of six-month administration of 325 mg/day of aspirin were evaluated in postmenopausal women. Although it had enough power to detect a difference of 15–17%, there were no significant changes in estradiol levels between the aspirin and placebo groups. On the contrary, administering a 6-week course of 100 mg/day of aspirin in our study caused a significant difference of 23% in the estradiol levels between the aspirin and placebo groups. Additionally, a cross-sectional study [23] found that NSAID (including aspirin and nonaspirin NSAID) users had significantly lower serum estradiol levels (i.e., ~ 16%). This study could not assess the duration of NSAID use or dosage information. Furthermore, summarized results of a recent study revealed that more frequent consumption of NSAIDs (aspirin, nonaspirin NSAIDs, or both aspirin and nonaspirin NSAIDs) in postmenopausal women (with or without BC) decreased circulating estrogens and estrogen metabolites [34]. When the association between aspirin use and estrogen levels was stratified by BMI, the results were sparse. A study found that BMI had no effects on the results [11], whereas some studies did not assess [22, 23] or had no clear pattern of interaction with BMI [17], or data were not available [34]. Major limitations encountered in these studies contain unavailability of information on the exact frequency of use or dose of aspirin or each NSAID, differences in the definitions of aspirin exposure, measurement of estrogen levels in only one time-point, lack of tissue-specific outcomes, and lack of certainty on the role of confounding factors in lowering estrogen levels.

On the other hand, a landmark meta-analysis [14] proposed no evidence to support that higher doses or longer durations of NSAID use were associated with a more significant reduction in BC than any intake, especially aspirin and ibuprofen use. Moreover, a cohort study [35] discussed that the association between NSAID use and BC might not be linear, and there was a possible U-shaped dose-response relationship between them. Indeed, the use of low-dose aspirin at 4+ days/week over 10 years was associated with a decreased risk of BC, while more frequent use of high-dose aspirin was associated with an increased risk.

In concordance with this U-shaped dose-response relationship, we observed that the reduction in estradiol levels was further in the initial time of aspirin administration. However, based on previously reported findings of six-month administration of 325 mg/day of aspirin [11], this effect may be gradually diminished over time and did not reduce estrogen levels which may conceal a more substantial effect at high doses and longer duration of aspirin use. There are several possible explanations for this inconsistency. First, the effects of aspirin on lowering estrogen levels may be significant only in the short term. Nevertheless, in the long-term use, the preventive effects of aspirin on BC may be attributed to the other pathways [24, 26, 35], and they are probably more important than their effects on estrogen levels. Second, given that estrogen levels were significantly higher in BC tissue than in plasma and that local estrogen production plays an essential role in the proliferation of invasive breast cancer cells in postmenopausal women [19], in the long term, locally chemopreventive effects of aspirin on breast tissue [30] may be more important than those on circulating levels [11]. Third, the importance of measuring estrogen metabolites in BC has received less attention. If we assume that the primary mechanism of aspirin is inhibition of the production of inflammatory prostaglandins and consequently aromatase production [4, 22, 35], the exploration of the roles of aspirin on pathways of estrogen metabolism should also be prioritized for investigation. Last, differences in individual characteristics and dietary factors [36, 37], multivitamin use [35], and genetic polymorphisms of the COX-2 gene [38] may affect estrogen levels in these studies and should be taken into account when the aspirin effects are evaluated.

The strength of this study includes the double-blind placebo-controlled randomized design and the high degree of medication adherence. The researchers implemented this study in a controlled design to increase response rates by incorporating promised incentives into their efforts, sending reminders by text messages and phone calls, and assuring participants protect their confidentiality and anonymity. Despite this, the non-response bias may have happened, wherein 69 (55.64%) participants became lost to follow-up between randomization and analysis. At the time of refusal, dropouts were asked to state their reasons. The major concern was the response load imposed by the study (i.e., length and frequency of participation). Since the non-respondents did not revisit for their follow-up, it was not clear how much their GI complications had led to non-adherence to the treatments or their reluctance to measure the serum levels, and also their statements about their complications were self-reported and could not be clinically validated. Other limitations include the lack of measuring estrone and androstenedione levels [17], the insufficient sample size to adjust covariates (e.g., BMI) and to investigate testosterone levels, relatively short follow-up period, concurrent use of other drugs or NSAIDs, and exclusion of premenopausal women. Therefore, the generalizability of this study is limited by the characteristics of our study population and the study setting.

## Conclusion

In summary, aspirin at low doses may reduce serum estradiol levels in postmenopausal women, supporting the growing evidence about aspirin use and decreased BC incidence. Nevertheless, a larger sample size and long-term follow-up with measurements of estrogen levels and its related compounds at different time points are needed to confirm our findings. In addition, further studies are needed to assess the effects of different doses and durations of aspirin and other NSAIDs on circulating estrogens and breast tissue locally. It should be clarified that the lower risk of BC in long-term aspirin users is due to decreasing estrogen levels, or there are other mechanisms in these people.

## Data Availability

The datasets used and/or analyzed during the current study are available from the corresponding author on reasonable request.

## Abbreviations

BC: Breast Cancer
BMI: Body Mass Index
COXs: Cyclooxygenases
NSAIDs: Non-Steroidal Anti-Inflammatory Drugs
PGE-2: Prostaglandin E-2
PPI: Proton Pump Inhibitor
SHBG: Sex Hormone-Binding Globulin
RCT: Randomized Clinical Trial
